# Comparing Pixel and Object-Based Approaches to Map an Understorey Invasive Shrub in Tropical Mixed Forests

**DOI:** 10.3389/fpls.2017.00892

**Published:** 2017-05-31

**Authors:** Madhura Niphadkar, Harini Nagendra, Cristina Tarantino, Maria Adamo, Palma Blonda

**Affiliations:** ^1^Ashoka Trust for Research in Ecology and the EnvironmentBangalore, India; ^2^Manipal UniversityManipal, India; ^3^School of Development, Azim Premji University, PES Institute of Technology CampusBangalore, India; ^4^Institute of Atmospheric Pollution Research, National Research CouncilBari, Italy; ^5^Institute of Intelligent Systems for Automation, National Research CouncilBari, Italy

**Keywords:** remote sensing, *Lantana camara*, object-based, tropical, understorey shrub, hierarchical, Western Ghats

## Abstract

The establishment of invasive alien species in varied habitats across the world is now recognized as a genuine threat to the preservation of biodiversity. Specifically, plant invasions in understory tropical forests are detrimental to the persistence of healthy ecosystems. Monitoring such invasions using Very High Resolution (VHR) satellite remote sensing has been shown to be valuable in designing management interventions for conservation of native habitats. Object-based classification methods are very helpful in identifying invasive plants in various habitats, by their inherent nature of imitating the ability of the human brain in pattern recognition. However, these methods have not been tested adequately in dense tropical mixed forests where invasion occurs in the understorey. This study compares a pixel-based and object-based classification method for mapping the understorey invasive shrub *Lantana camara* (*Lantana*) in a tropical mixed forest habitat in the Western Ghats biodiversity hotspot in India. Overall, a hierarchical approach of mapping top canopy at first, and then further processing for the understorey shrub, using measures such as texture and vegetation indices proved effective in separating out *Lantana* from other cover types. In the first method, we implement a simple parametric supervised classification for mapping cover types, and then process within these types for *Lantana* delineation. In the second method, we use an object-based segmentation algorithm to map cover types, and then perform further processing for separating *Lantana*. The improved ability of the object-based approach to delineate structurally distinct objects with characteristic spectral and spatial characteristics of their own, as well as with reference to their surroundings, allows for much flexibility in identifying invasive understorey shrubs among the complex vegetation of the tropical forest than that provided by the parametric classifier. Conservation practices in tropical mixed forests can benefit greatly by adopting methods which use high resolution remotely sensed data and advanced techniques to monitor the patterns and effective functioning of native ecosystems by periodically mapping disturbances such as invasion.

## Introduction

Plant invasions occur at rapid rates within diverse habitats across the globe, from grasslands to dense forests, and spread over large areas (Mack et al., 2007). It is imperative for most owners and land managers to therefore keep track of newer invasions within their landscapes and continuously monitor existing ones so as to disallow further spread and intrinsic transformations within the ecosystems (Gardener et al., 2011; Dodet and Collet, 2012; Hiremath and Sundaram, 2013). Systematic and repeated observations – monitoring – are essential for ecosystem management to enable managers to detect, document, and respond to changes. Monitoring of the invasion process involves repeated observations for recording the advance of the invasion over different seasons and across years, which is made possible by synoptic and frequently repeated observations of large and small landscapes by remote sensing (RS). Mapping invasion over long temporal periods has been possible due to the now long history of the availability of RS data across many regions in the world (Laliberte et al., 2004; Gavier-Pizarro et al., 2012).

Recent advances in RS technology now provide for very high resolution [VHR – multispectral resolution 2 m or lower as stated in Nagendra and Rocchini (2008)] data for land cover mapping. The use of these data for remote detection of invasive plants has proved useful in several different ecosystems, and for a variety of species (Mohamed et al., 2011; Doody et al., 2014; Niphadkar and Nagendra, 2016). Comparative studies of VHR data and medium resolution data for invasive species detection have often proved that VHR data were extremely effective (Everitt et al., 2008). However, VHR data are increasingly proving to be of much use in the process of invasion mapping specifically because of the precision and detail that these data provide in separating signatures of different land cover types. These data have been used largely in temperate regions as well as grasslands to single out invasion by one or two species occurring in homogeneous stands (Laliberte et al., 2004; van Lier et al., 2009; Marshall et al., 2014). Although VHR data is now widely being used for invasion mapping, many scientists argue that the amount of detail provided by these data may sometimes prove to be too much for easy separation of intended classes (Nagendra and Rocchini, 2008). The amount of complexity that these datasets provide is such that regular parametric approaches to image classification may not be sufficient to derive maximum benefits from the large amount of information they contain. Newer methods such as object-oriented approaches (Lantz and Wang, 2013), Support Vector Machines (Gil et al., 2013), and Spectral Angle Mapper (Schmidt and Witte, 2010) provide the facility to utilize not only the spectral information, but also the spatial and contextual information in VHR data to characterize landscapes in their complexity, and pick out the invasive species much more efficiently.

Object-based image classification is emerging as an effective tool for identifying land cover types that are identified as independent units, not only on the basis of spectral information from the pixels, but also from spatial information that is inherent in the structure of the land cover type (Yu et al., 2006). Many land cover types are observed to appear in a particular pattern in a satellite image, within the type, as well as with reference to co-occurring types or landscape features. This information relevant to their spatial characteristics – such as shape, compactness, proximity – can be included in object-based classifiers, identifying patches of each land cover class as independent objects conforming to these characteristics. This approach has been shown as an effective mechanism for using an ecological approach to habitat mapping using multi-date VHR data across multiple locations (Nagendra et al., 2015). Thus, landscape complexity, of spectral as well as spatial and contextual information, can be included in the object-based approach to land cover classification. This approach has proved useful in mapping invasive plant species in recent years (Brenner et al., 2012).

The spatial resolution of VHR data allows for this complex object-based approach to be used in detection of invasive plant species which occupy contiguous patches when they occur in clumps (Wan et al., 2014). The combination of using VHR data in an object-based approach further augments mapping capabilities provided by RS technology, utilizing the enhanced spatial detail from the VHR data along with the capacity of object-based mapping to separate independent patches of invasive alien plants (Lantz and Wang, 2013; Wan et al., 2014).

The remote detection of invasive plant species in tropical forests has been an uphill task primarily because the diversity of tropical forests manifests in a large amount of spectral variability shown by the species themselves, and because of the intricate mixed species-community complexes that are formed when several species occur together in multi-species stands. However, recent advances in technology such as hyperspectral RS in combination with VHR data on airborne platforms provide cutting edge mechanisms for detecting invasive plants (Asner et al., 2008b). These studies have used fine resolution spectra to map out individual tree species in tropical forests, using their phenological or structural traits to single them out from the dense mixed top canopies. While invasive tree species (Asner et al., 2008a,b) and aquatic species (Underwood et al., 2007) have been easily detected using VHR data, cryptic understorey species in forests have definitely proved harder to detect. Large, open, pure, and continuous stands of invasive shrubs have been easier to map, in temperate regions (van Lier et al., 2009; Shouse et al., 2013), as also in tropical forests (Taylor et al., 2010; Padalia et al., 2012), proving it more convenient for managers to mitigate this threat. However, a considerable proportion of invasive plant species that occur in tropical forest landscapes are shrubs which appear in the understorey (Richardson and Rejmanek, 2011), covered by the canopy of the overstorey tree species which themselves occur in several tiers. This makes it even more difficult to be able to map the understorey invasive plants as the satellite sensor cannot detect their presence underneath the multiple tiers of canopy above [see Joshi et al. (2004) for a summary of RS for invasive species’ canopy characterization according to life forms and Niphadkar and Nagendra (2016) for a summary of RS mapping using functional traits of invasive plants]. It is imperative that some mechanisms of detection and mapping understorey invasive shrubs are devised which would help in their periodic monitoring toward possible prevention from further spread.

Given the capabilities of object-based approaches for mapping contextual cover types, and those of VHR data for detection of detail in the landscape, a combination of these tools seems like an effective methodology to be followed for remote detection of invasive plants in the understorey of tropical forests. However, very few such studies have been undertaken which incorporate both, the method and the data type, to detect tropical understorey invasion (Ghulam et al., 2014). Even as object-oriented techniques have been used for mapping invasion in the tropics (Walsh et al., 2008), their use for understorey shrub detection has been limited. With this perspective, mapping the distinctive clumping behavior of understorey invasive shrubs can be made possible by using these capabilities of the object-based approach as applied to the VHR data which can characterize their complexity.

Several studies have proved that in addition to using direct reflectance from species, using indices of various kinds generated from the reflectance bands has also been successful in marking out invasive species (Karnieli et al., 2013). This has been effective using medium spatial resolution satellite data not only for large stands (Sridhar et al., 2010), and open area- (Zimmermann et al., 2011) or wetland-species (Ouyang et al., 2013), but also for understorey species such as the Amur honeysuckle *Lonicera maackii* (Wilfong et al., 2009; Johnston et al., 2012). While many detection techniques use vegetation indices directly as bands for classification or in regression with field observations (Johnston et al., 2012), some studies have also indicated the effective use of index thresholding with satellite data (Bhandari et al., 2012) for delineating land cover classes or even invasive grasses (Olsson et al., 2011).

The tropical forests of the Western Ghats are home to a great diversity of plant species comprising different life forms, forming complex vegetation assemblages. Reports on plant invasion in the Western Ghats have emerged in the recent past (Muniappan and Viraktamath, 1993), wherein large tracts have been covered by unchecked spread of invasive shrub species (Bhagwat et al., 2012; Sundaram and Hiremath, 2012). While documentation of invasive species occurrence and spread is growing (Chandrasekaran and Swamy, 2010; Kimothi and Dasari, 2010; Rao and Sagar, 2012; Joshi et al., 2015), few studies have undertaken invasive species mapping using RS technology in the Western Ghats (Prasad, 2008).

Among these multiple invasive alien plants, *Lantana camara* (henceforth, *Lantana*) – a tropical woody shrub native to Central and South America – is particularly aggressive and has established itself rapidly across tropical and sub-tropical regions on several continents (Bhagwat et al., 2012). *Lantana* has been reported from several forest types across the tropics (Islam et al., 2001; Raghubanshi and Tripathi, 2009)). Recent studies in India report an almost 10-fold increase in abundance of *Lantana* at a specific site in the Western Ghats (Sundaram and Hiremath, 2012). At such high rates of spread, it is extremely essential to develop potential methods of monitoring the spread regularly in areas where increase has been particularly abundant.

We seek to describe and compare two RS approaches used to find an optimum method for mapping the understorey invasive shrub species *Lantana camara* in the tropical dry forest landscape of the Biligiri Rangaswamy Temple Tiger Reserve in the Western Ghats region in southern peninsular India. Although high incidence and spread of *Lantana* has been reported from this site (Sundaram and Hiremath, 2012), mapping using RS technology has not been done for this species so far. We attempt to discriminate the invasive shrub within this forested landscape using a pixel-based and an object-based method in classifying multi-season VHR data, adopting a hierarchical framework in both approaches. We build on the response of the species to selected vegetation indices to delineate areas where *Lantana* occurs based on the dominant forest types where it is reported in the understorey.

## Materials and Methods

### Study Area and Vegetation Mapping

**FIGURE 1:**
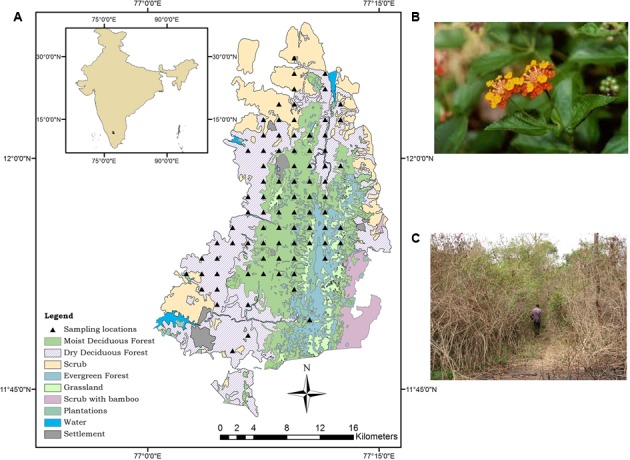
**(A)** Location of study area with sampling sites, ground truth sites and extent of satellite data coverage. **(B)** *Lantana camara* flower. **(C)** Dense thicket formed by *Lantana camara*.

The Biligiri Rangaswamy Temple Tiger Reserve (BRT TR) is a highly biodiverse protected forest situated at the convergence of the Western and Eastern Ghats in southern peninsular India (**Figure 1**). This distinctive location imparts a uniqueness to this 540 sq km protected forest in several ways. The BRT TR has a hilly terrain of ranges lying in the north–south direction, varying between 600 and 1800 m above sea level and receives rainfall from both the southwest and northeast monsoon winds, with an annual average ranging between 898 and 1750 mm depending on location within the sanctuary. The study area has mostly well-drained gravelly clay soils, classified as typic ustropepts (Anonymous, 1996). Temperatures range from 11°C in winter to 42°C in summer with an average of 25.3°C (Murali et al., 1998). No studies specifically on climate change have been conducted so far in this location. The complexity and diversity in the vegetation of BRT TR is a function of the spatial variability in topography and climate, along with human activities including fire. Using a RS technique of visual interpretation of SPOT data, the vegetation of the sanctuary has been classified into 10 different types from dry scrub thickets to dense wet evergreen forests at high elevations (Ramesh, 1989; Ramesh and Menon, 1997). The denser cover types among these support tiered forests, which are found in mixed stands. Almost 90% of forested area constitutes deciduous forests or thickets. A later study used 3-date NDVI and a rule-based tree model for classifying the vegetation of the BRT TR into 16 different classes, which included transition classes such as ecotones between moist and dry deciduous forest types (Krishnaswamy et al., 2004).

### Species

*Lantana camara* is a native of Central America, and was introduced into India by the British in 1809 (Cronk and Fuller, 1995; Bhagwat et al., 2012). In the BRT TR area, it was first reported in 1934 as an incidental record (Ranganathan, 1934). More recent studies have reported its occurrence (Murali and Setty, 2001), and subsequently its widespread increase (Sundaram and Hiremath, 2012). These studies provide important baseline information about invasion of *Lantana* in this landscape.

Due to its highly invasive nature and large area spread, techniques for mapping *Lantana* using RS technology have been developed and refined in other countries such as Australia (Taylor et al., 2010, 2012; Dlamini, 2012). However, although predictive modeling of *Lantana* spread has been done for locations across the Indian subcontinent (Priyanka and Joshi, 2013; Adhikari et al., 2015), there are very few examples in India where mapping of *Lantana* using RS techniques has been attempted (Prasad, 2008; Kandwal et al., 2009; Kimothi and Dasari, 2010). This work differs from these earlier mapping efforts mainly in three ways – the use of multi-season VHR data, the implementation of a hierarchical classification approach, and the use of an object-based classification technique. Within this framework, we also utilize image transformations such as texture and vegetation indices to help discriminate *Lantana* from surroundings.

### Field Work

Due to the highly expensive nature of VHR data, it was possible to acquire VHR satellite imagery for only a 100 sq km patch within the BRT TR. Systematic sampling for presence of *Lantana* was conducted in this 100 sq km patch consisting of all major land cover types. The sampling methodology allowed for detailed data collection at 35 locations in the 100 sq km covered by the satellite images. These sites were located as center points of a 2 km by 2 km grid covering the study site. In order to make sure to include disturbed areas where a greater presence of invasive species was likely, additional locations were identified along roads and human settlements for detection of *Lantana*. In all, ground truth data were collected for 115 locations (with a high-precision GPS, data recorded up to six digits precision) within the area covered by satellite data. This included detailed sampling for the 35 sampling locations, and ground truth on presence/absence of *Lantana* for the remaining 80 locations (**Figure 1**). The sampling involved laying out of plots 80 m by 5 m across. Within these plots, 5five different locations separated from each other by 20 m were reached, and at each location *Lantana* presence or absence was noted by surveying an area within a 5 m diameter. Surrounding forest type was also noted for a better understanding of the land cover in order to aid in the supervised classification approach. Detailed sampling for the surrounding forest type using complete enumeration of trees and shrubs >10 gbh has already been done in an earlier study within the same plots (Sundaram, 2011). This information was used for understanding the cover type and also used in classification later.

### Data

**FIGURE 2:**
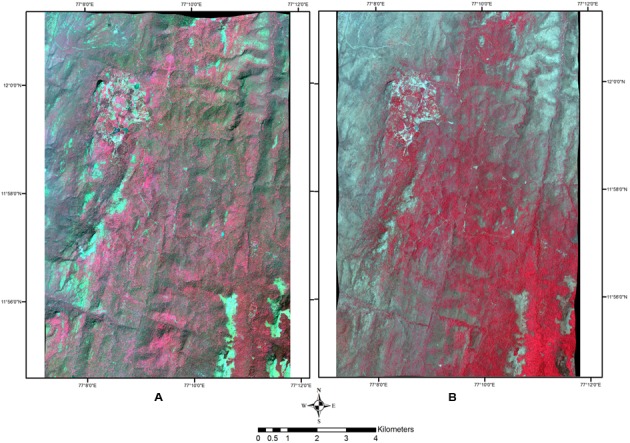
Satellite data used. **(A)** Wet season image – January 7th, 2011. FCC with R: 4 (NIR); G: 3 (Red); B: 2 (Green). **(B)**. Dry season image – March 14th, 2013. FCC with R: 7 (NIR1); G: 4 (Red); B: 3 (Green).

The 100 sq km area covered by the available satellite data was selected on the basis of the number of different forest types it supported, as this would allow mapping of *Lantana* in all the diversity of habitat types that supported its growth and proliferation. We used a 4-band multispectral image acquired on January 7th, 2011 by the Geo-Eye satellite as the wet season image (spatial resolution 2 m, image acquisition time 10:47 local time IST), and an 8-band multispectral image acquired by WorldView 2 (spatial resolution 2 m, image acquisition time 11:26:27 local time IST) on March 14th, 2013 as the dry season image (**Figure 2**). The unique rainfall regime experienced by this site allows for only a short window of time when it is completely dry, and hence acquisition of cloud-free satellite data for this time was particularly challenging.

### Image Pre-processing

Satellite data analysis involved two stages, the first of which was pre-processing to make them usable for further analysis. For pre-processing, both images were first ortho-rectified in ERDAS Imagine TM software to a sub-pixel accuracy. The images were then co-registered to each other. Top of atmosphere reflectance values – reflectance data measured by the sensor from its location above the atmosphere – were then used in all further processing of the data.

#### Texture Layer

Since *Lantana* is a shrub with small leaves about 2–10 cm long, and thorny stems, forming clumps or thickets within forest understorey, its general structure would be differentiable within the image if the pattern of spectral response is distinguishable from that of the overstorey canopy. Texture measures can prove useful in vegetation discrimination especially for VHR data (Ge et al., 2006), as the high spatial resolution produces quick changes in the spectral pattern variation among adjoining pixels (Hudak and Wessman, 1998). Kimothi and Dasari (2010) have effectively used texture measures along with image fusion techniques for mapping *Lantana* in the Sivalik region.

We tested several statistically derived texture measures such as variance, skewness, and entropy. Variance measures the local variance within the processing window defined by the user, and replaces the central pixel with that value. Skewness is a measure of the asymmetry of the data around the mean of all pixel values within a window. Entropy is a measure of randomness within a measurement window. Texture images were developed in the software ENVI and then imported into eCognition. Each of the measures was tested with differing window sizes and parameters. The coefficient of variation (CV) for each image was obtained for these indices and compared. The index with the lowest CV would help identify the measure with low variation, yet be able to characterize *Lantana* as it structurally appears on the ground. Among these measures, the CV for entropy was the lowest (**Table 1**). As the image has a very fine spatial resolution of 2 m, the window size of 7 × 7 pixels for performing texture analysis appeared to perform well enough to separate different textures for the land cover types. This was observed with reference to the size and appearance of the *Lantana* clumps on the ground, wherein the texture measure was able to characterize the patches most efficiently. Entropy was calculated at different window sizes and compared visually for discriminatory ability. Kernel windows of smaller size produced images that were too grainy to separate different land cover classes by textural differences. An entropy layer using a 7 × 7 pixel window was therefore finally considered appropriate for both the images.

**Table 1:** Coefficient of variation within image for different texture measures at different window sizes.

Texture measure	Window size	Wet season image	Dry season image
Entropy	3	0.468	0.313
	5	0.255	0.236
	7	0.2	0.188
Variance	3	4.296	6.369
	5	3.631	5.138
	7	3.348	4.798
Skewness	3	-7.219	-14.710
	5	-4.053	-9.609
	7	-3.629	-9.217

### Image Analysis for Detection of *Lantana*

#### Preliminary Exploratory Analysis of Satellite Data for Bands and Indices

We did a simple correlation analysis to look at band correlations for each image separately, to check for redundancy of information in bands that were highly correlated. *Lantana* is a species that largely occurs in open forests and performs well in high light conditions. It has a high tolerance range for moisture availability and survives in very dry as well as moist conditions (Day et al., 2003). Previous studies (Kandwal et al., 2009; Kimothi and Dasari, 2010; Taylor et al., 2010) have indicated the effective use of vegetation indices for detection of *Lantana* in hilly areas as well as forested regions. Kandwal et al. (2009), have tested the use of 29 different indices for detection of *Lantana*, concluding that SAVI was the most effective in their study site in the Himalayan forests. Based on these studies, we calculated a set of five indices (Normalized Difference Vegetation Index, Soil Adjusted Vegetation Index, Transformed Vegetation Index, Green–Red, and Blue–Green–NIR) for each of the two season images (Refer to Supplementary Table 1 for formulae of indices used).

#### Preliminary Exploratory Analysis of *Lantana* Presence Using Logistic Regression

A logistic regression based model was considered as a suitable method for identifying the reflectance bands and indices that were more indicative of the species’ presence. Logistic regression allows the calculation of a multivariate regression between a dependent variable which is binary, and a set of several independent variables. It is particularly relevant in this analysis as we were trying to understand if the spectral response recorded by the sensor has been able to detect the presence/absence of the invasive species strongly enough to have predictive power. We used the presence/absence status of *Lantana* from 115 locations (50 for model training and 65 for testing) in the 100 sq km area of the field site which was covered by the remotely sensed data. Each of the two different seasonal images (four bands for wet – Geo-Eye image and eight bands for dry – WorldView 2 image) were considered separately, and then together as a 12-band image, in which all bands from both seasons were considered as predictor variables. Since the data were binary, we developed generalized linear logistic regression models with a binomial distribution function using the reflectance bands and five indices, separately for the independent seasonal images, and the 12-band image. No comparative analysis of the complete models was done; the reflectance bands and indices were used independently in further classification. All statistical analysis was done in open access software R (Package ‘car,’ function ‘glm’) (R Core Development Team, 2012).

#### Pixel-Based Classification

A standard pixel-based analysis using maximum likelihood supervised classification was performed at first to check the efficiency of VHR data in detecting invasive understorey species. A hierarchical approach was undertaken in which the image was first classified into basic land cover classes, and then a select few of these classes were further processed for detecting *Lantana*. Since *Lantana* is an understorey species, it has always proven difficult to map out separately from the overstorey canopy vegetation, unless it occurs in continuous open patches within the forested area. Hence, it was important to separate out the different vegetation types that support this species, and then apply further processing to these classes.

Supervised classification was performed separately on the images for the single wet season image, and the combined layer stack of wet and dry season images, with and without texture bands. Signatures were identified from known locations for nine different cover classes in the scene. These included Water, Shadow, Bareground, Impervious, Scrub, Dry Deciduous Forest, Moist Deciduous Forest, Evergreen Forest, and a pure class for the invasive species *Lantana*. After detailed evaluation of the signature set, a supervised classification was performed using the maximum likelihood method. As *Lantana* forms the understorey vegetation cover, only those vegetation classes which yield to detection of vegetation under the top canopy were considered for further analysis. These included Moist Deciduous Forest, Dry Deciduous Forest, Scrub and the *Lantana* class (with pure *Lantana* stands). A mask (0 = absence, 1 = presence) was generated using these four classes. The mask of selected vegetation classes was then applied to the different index images generated from mathematical transformations of bands (mathematical transformation formulae listed in **Table 2**), to mask out classes with no probability of occurrence of *Lantana*. Of the five indices that were tested in the exploratory analysis, only three were chosen to be included in this analysis. NDVI was excluded as it mostly corresponded to the overstorey canopy response. TVI and NDVI were very closely correlated (Pearson’s *K* = 0.99 for both dry and wet season indices). The resulting images obtained from masking were then subjected to independent thresholding of their values. The thresholding was based on a range defined by observed iterative checking for index values at occurrence locations of *Lantana* from field observations. A set of 45 field data locations were used for defining the thresholds of indices with presence of *Lantana*, while the rest (50) were used for testing. **Table 2** indicates the thresholds decided upon for each index after exploratory testing.

**Table 2:** Maximum and minimum values of indices defined for thresholding.

	Original values – wet		Original values – dry		Thresholds set	
Index	Minimum	Maximum	Minimum	Maximum	Minimum	Maximum
SAVI	-0.357	1.7555	0	1.7727	1.36	1.71
Green-Red	-505	576	-70	49	120	175
Blue-Green-NIR	150	527	43.013	345	268	312

#### Object-Based Classification

The object-based approach to classification was conceptualized with the structural and spatial behavior of the invasive species in focus. Such a technique has been proved an effective mechanism for using an ecological approach to mapping anthropogenic pressures using multi-date VHR data across multiple locations (Nagendra et al., 2015). As the species spreads in large contiguous clumps in open areas, as well as under somewhat closed canopies, its spatial form and appearance may be easily detected by considering spatial characteristics along with spectral values of reflectance, identifying these as independent objects in a satellite image (for e.g., Yu et al., 2006).

**FIGURE 3:**
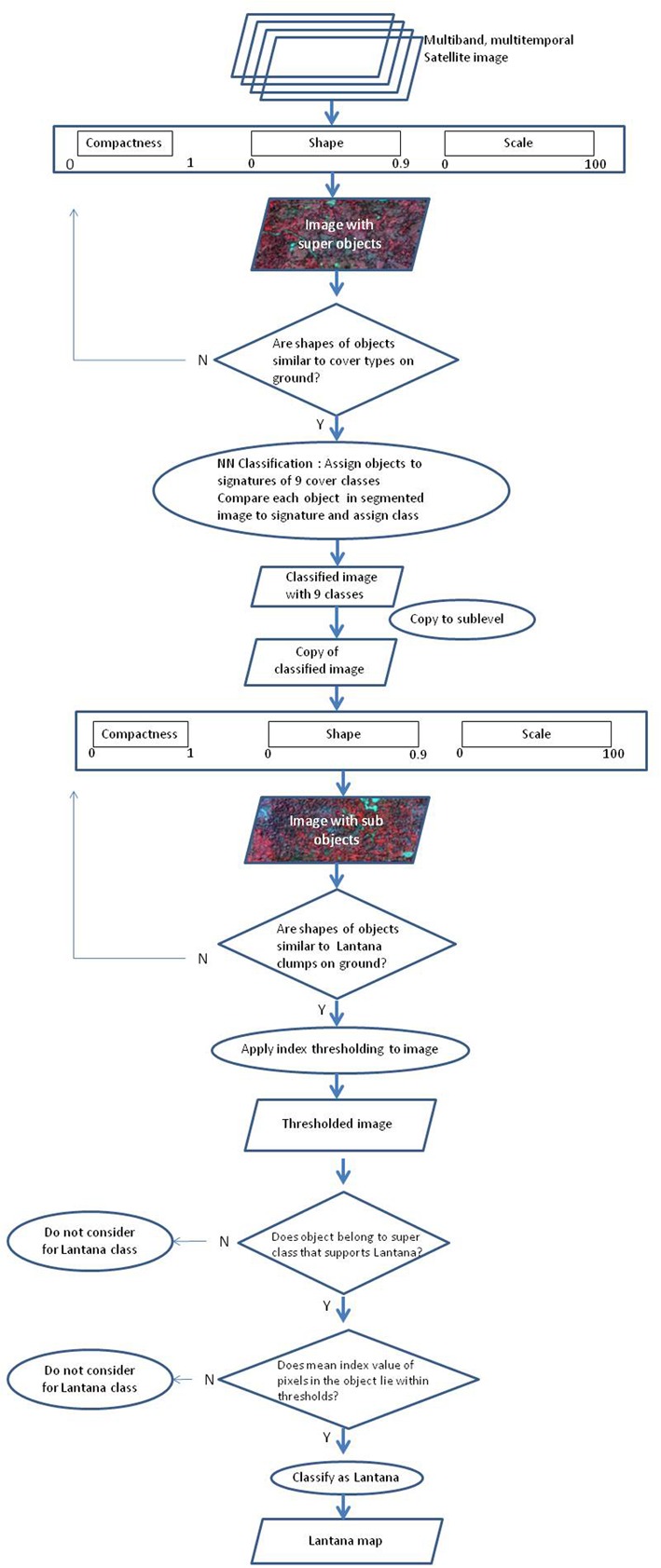
Flowchart indicating the process of object-based classification to obtain a map for *Lantana* spread.

An object-based method of segregating pixels into classes replicates the elements of human method of interpretation of satellite data. A hierarchical object-based approach was developed for delineating clumps of the invasive species within the image. eCognition TM software was used for all the object-based analysis. In an object-based approach, the most important step is to perform a good segmentation of the image such that the objects are delineated clearly to characterize the landscape for the specific objective of interest. Segmentation is a process by which, using multiple spectral, spatial and contextual parameters, entire images are subdivided into objects at various levels - from the pixel level to groups of pixels as larger and larger clumps at different scales, up to the entire image level. The following methodology (**Figure 3**) was followed for each of the wet season image and the wet-and-dry stack image, each one with, and without, the texture layer added as input for the segmentation process.

First, a multi-resolution segmentation was performed to delineate objects which represented different land cover classes from the image. It is difficult to find a certain agreement in literature for setting optimum values for the parameters for segmentation (Kavzoglu and Yildiz, 2014). eCognition uses a hierarchical approach for classification (**Figure 3**), wherein segmentation results are stored at ‘levels’ above or below each other; objects are termed sub-objects or super-objects depending on their size and the level at which they are stored. The lowest level is usually the single pixel-level, while the highest level is that of the entire image. The segmentation was performed after setting up the parameters of compactness, scale and shape for object demarcation. The scale parameter is used to determine the maximum allowed heterogeneity in the image objects. Thus, for a given scale parameter, a heterogeneous image will yield smaller objects than a homogeneous image (eCognition User Guide). Shape and compactness are parameters used to define the homogeneity of the objects. The shape parameter (ranging from 0 to 0.9) is used to define objects with spatial homogeneity, at a cost to spectral homogeneity. Lower shape values indicate more percent weight given to spectral values of pixels in the data for defining objects. Compactness (ranging from 0 to 1.0) identifies the compressed nature of the objects, based on weak spectral contrasts, and is used to optimize objects for their compact shape. For super objects, which are large in size relating to land cover types, a setting of 0.03 for compactness (less compact), a shape parameter of 0.01 units (complexity of shape) and a scale of 100 units (larger size) was considered appropriate for delineating clumps of the size that are regularly seen for this shrub in the field site. The objects generated by our segmentation process were stored at a level we defined as ‘Large_objects’ which would be subjected to further segmentation in a later process. Next, these objects were copied to two new levels called ‘Samples’ and ‘Classification,’ below the current level. This was done so that the objects identified in the segmentation could be used in the classification process as separate samples or signatures and the classification would be stored in a different level. Next, a supervised classification approach was implemented in which specific objects delineated in the segmentation process were assigned to land cover classes to be identified as ‘signature objects.’ Land cover classes to be classified were defined in the Class Hierarchy window. Here, the defining feature statistics of each class (defined by eCognition TM as ‘Nearest Neighbor membership functions’) which would be used for classification were specified. These were taken as the mean values for Red and Green bands, as well as the Green–Red and Blue–Green–NIR index. Based on field information, objects that belonged to particular classes were selected and assigned to those class signatures defined in the Class Hierarchy, in order to designate them as ‘signature objects’. Sample statistics were therefore taken from these object samples.

Next, a nearest neighbor classification was performed on the objects, assigning each of them to appropriate classes based on the statistics defined in the Class Hierarchy. The nearest neighbor classifier algorithm in eCognition searches for sample image objects that are closest to the feature space characteristics of each segmented object, assigning the objects to the closest classes with similar feature space characteristics. The image was classified into nine land cover classes based on object signatures, identical to the ones in the pixel-based classification. These were – Scrub, Moist Deciduous Forest, Dry Deciduous Forest, Evergreen Forest, Shadow, Water, Impervious, Bareground, and a separate class Lantana (for independent separable pure clumps of *Lantana*). These classes were then copied to a new map, and only the classified object level was retained. Next, for the classes that had high probability of occurrence of *Lantana* in the understorey, as well as the pure Lantana class, a multi-resolution segmentation was performed with modified parameters. This process was implemented to delineate smaller objects within the larger objects of forest canopy classes identified at the higher level. Parameter values were tested, and objects delineated were visually assessed. If the objects appeared unrealistically small or large compared to *Lantana* clumps in the field, these parameters were rejected. The parameters arrived at for this segmentation producing the sub-objects (criteria: smaller size, more compact, complex shape) were: scale = 25, compactness = 0.4, and shape = 0.2, which clearly delineated pixel clusters forming objects that were similar to those observed in the landscape for clumps of the invasive shrub. The smaller objects were saved at a hierarchical level lower than that of the large objects. Finally, objects were classified into the *Lantana* class based on index-image thresholding. Thresholds were obtained from a preliminary exploratory analysis of objects that had mean values of each index. Class rules were defined such that objects would be classified into the *Lantana* class only if (a) the mean index value per object lay between the defined thresholds, and (b) if they belonged to the set of super-classes identified to be the ones supporting understorey *Lantana*. Here, too, the standard nearest neighbor statistics for classification were incorporated into the class rules. (See **Figure 3** for complete process).

#### Accuracy Assessment

Accuracy assessment for each of the two methods of classification was performed in the software IDRISI. Ground truth presence/absence information from the 115 field observation points was used as validation data. Each of the classified maps obtained from the three different indices, for each of the classification methods – pixel-based and object-based was tested for accuracy and confusion matrices were obtained.

## Results

### Logistic Regression

The results of the logistic regression analysis for each of the wet season and wet-and-dry season stack images were not conclusive. Overall, one could not draw confirmatory conclusions regarding the particular reflectance bands or indices that contributed in a large way to helping to distinguish the presence of *Lantana* in the field. Hence, it seemed appropriate to use all reflectance bands and indices for pixel-based and object-based classification as next steps. Since the TVI, NDVI, and SAVI were very highly correlated with each other (Pearson’s *K* = 0.99), it was decided to use the indices SAVI, Green–Red, and Blue–Green–NIR index – which were relevant in this landscape, for further mapping *Lantana*.

### Pixel-Based Classification

**FIGURE 4:**
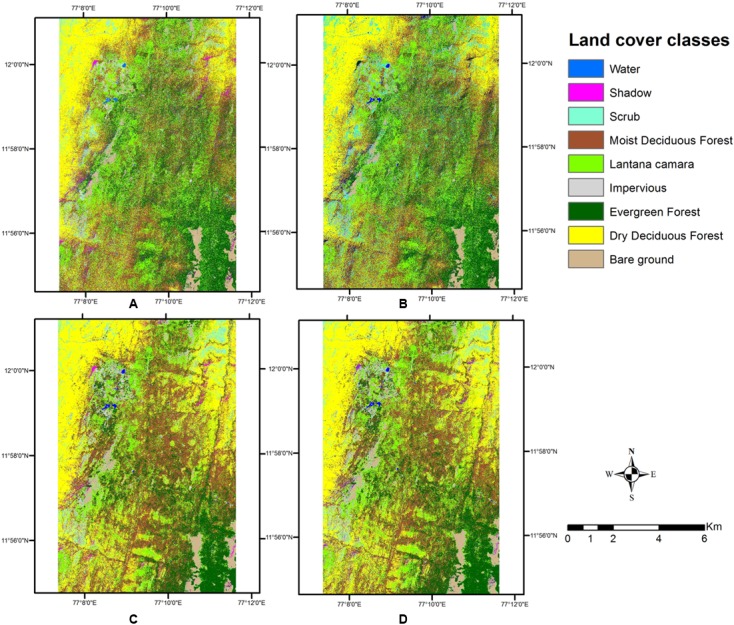
Land cover types obtained from pixel-based classification. **(A)** Wet season, with texture. **(B)** Wet season, without texture. **(C)** Two seasons, with texture. **(D)** Two seasons, without texture.

The pixel-based classification produced very different results for the two different image stacks, although the overall classification results appear visually similar (**Figure 4**). Area calculations for each cover type did vary across the methods of classification (**Table 3**).

**Table 3:** Areas under different land cover types for the different methods of classification (sq km).

	Wet season				Two-season			
	With texture		Without texture		With texture		Without texture	
Classes	Pixel-based	Object-based	Pixel-based	Object-based	Pixel-based	Object-based	Pixel-based	Object-based
Bareground	4.494	2.185	4.428	3.098	4.684	2.014	4.638	2.794
Lantana	18.357	9.250	19.229	6.566	14.770	7.806	16.520	5.566
Evergreen Forest	18.200	12.046	17.332	16.916	13.738	11.154	13.691	20.921
Dry Deciduous Forest	18.537	19.938	19.764	23.007	24.810	21.057	25.352	22.577
Moist Deciduous Forest	25.836	46.955	24.731	39.403	29.065	48.225	26.649	36.923
Scrub	6.739	3.696	6.296	5.296	5.678	3.941	5.601	5.590
Water	1.033	0.218	1.350	0.005	0.500	0.146	0.583	0.034
Shadow	0.969	0.114	1.090	0.041	0.733	0.115	0.901	0.000
Impervious	0.388	0.120	0.333	0.218	0.573	0.087	0.618	0.146
Unclassified	0.040	0.031	0.040	0.003	0.040	0.008	0.040	0.000
**Totals**	**94.592**	**94.552**	**94.592**	**94.552**	**94.592**	**94.552**	**94.592**	**94.552**

The single wet season classified image created a salt and pepper appearance, as the classes were not identified as contiguous sets of pixels. Several pixels or small groups of pixels appeared interspersed with each other, in a spatially disjunct manner. The two-season combined image, however, shows spatially contiguous classes and less of scattered classes in the image. Some misclassifications in the classes of Scrub and Dry Deciduous Forest seem to have occurred. Incorporating the texture layer in the pixel based classification did not improve much upon accuracies, but a visual inspection of the classified map indicates how clearly the contiguity of classes is maintained in this output. The texture measure helped to keep the spatial integrity of the classes, and thus clumps of pixels emerged as objects in the output. As *Lantana* shows clumping behavior in the areas it occupies (for e.g., Ramaswami and Sukumar, 2013), and this clumping allows it to spread further in dense thickets (Walton, 2006), it is imperative that this behavior is brought out by the mapping mechanism.

This classification of land use classes at a preliminary level was used to create a mask of four different classes, which was then used to perform thresholding on indices to separate out *Lantana*. The variation in the outputs of the first level classification did allow for different outcomes at the second-level of classification. However, index-based thresholding produced very similar maps of *Lantana* for all indices (**Figures 6D–F,J–L**) as this was done only within the already identified specific vegetation classes, and thresholds applied were the same across all the first level outcomes. Results from SAVI, however, did leave out clumps in the Northwestern corner of the image.

**FIGURE 5:**
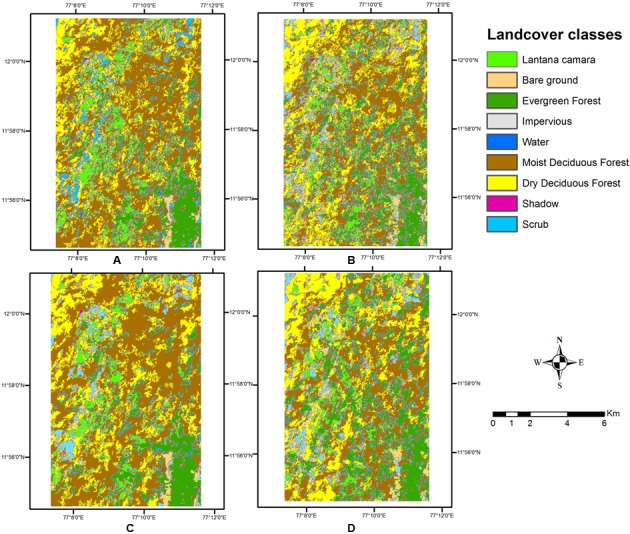
Land cover types obtained from object-based classification. **(A)**. Wet season, with texture. **(B)** Wet season, without texture. **(C)** Two seasons, with texture. **(D)** Two seasons, without texture.

There was not much difference in the results of area under *Lantana* after index thresholding with the addition of the texture band (**Table 4**), as is visually evident in the **Figure 5**.

**Table 4:** Area under *Lantana* based on pixel-based index thresholding (sq km).

	Two-season		Wet season	
	With texture	Without texture	With texture	Without texture
SAVI	34.786	34.861	33.532	33.959
Green–Red	40.372	40.475	40.088	40.573
Blue–Green–NIR	35.520	35.616	37.739	38.020

### Object-Based Classification

Object-based classification produced fairly consistent results for both types of classification (**Figure 5** and **Table 5**). The exploratory work in scale, shape, and compactness criteria for the segmentation helped in identifying objects that were realistic in their appearance to clumps of the invasive shrub found in the field. The object-based method was able to pick out the thicket-forming behavior of *Lantana* specifically due to the delineation of contiguous objects using both spectral and spatial information from the image. On visual inspection, the wet-and-dry-season image appeared to have fared better in terms of classifying contiguous areas for each class, as well as identifying smaller clumps interspersed between higher level classes for *Lantana* as a pure class.

**Table 5:** Area under *Lantana* based on object-based index thresholding (sq km).

	Two-season		Wet season	
	With texture	Without texture	With texture	Without texture
SAVI	35.573	30.733	35.676	32.792
Green-Red	40.471	35.097	40.210	36.884
Blue-Green-NIR	37.414	33.121	38.437	35.315

**FIGURE 6:**
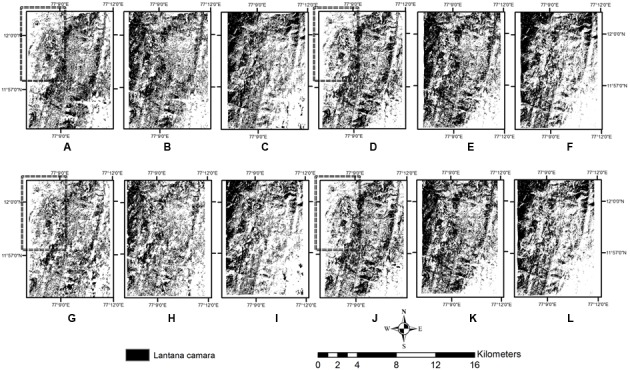
Comparison of *Lantana* mapped with thresholding of different indices with object-based **(A–C,G–I)** and pixel-based classification **(D–F,J–L)** using two season data. **(A,D,G,J)** are obtained from SAVI, **(B,E,H,K)** are obtained from Green–Red index, and **(C,F,I,L)** are obtained from Blue–Green–NIR index. **A–F** are obtained with the use of texture, **G–L** are obtained without texture. Rectangle highlights area where *Lantana* was undetected using SAVI.

Selected classes from these classified maps were further subjected to multi-resolution segmentation and thresholding based on indices (**Figures 6A–C,G–I**). In the SAVI image, *Lantana* was not classified at the north–west corner of the image, while both the other indices identified objects in that area as *Lantana*, similar to that in the pixel-based classification. The Blue–Green–NIR index image seems to have overclassified several areas into *Lantana* which may not have the presence of the shrub in such abundance (**Table 5**).

### Accuracy Assessment

#### Overview

The accuracy assessment method used was the standard pixel-based presence/absence of *Lantana* compared in the classified maps to the reference field information. Overall accuracies for all the methods used hovered around 60% for the identification of *Lantana*. As an overview, the two-season image with the texture band seemed to perform well in terms of accuracy of mapping *L. camara*. User’s accuracies were generally higher than producer’s accuracies or overall accuracies for each map. Object-based accuracies were generally higher for both the wet season and two-season images, except in the case of the SAVI index for the classification with inclusion of texture layer, where the SAVI index-based classification showed a lower value.

#### Object-Based

Overall accuracy was highest in the classification produced by the Blue–Green–NIR index for the two-season image, using the texture band. Producer’s accuracy was highest for the Green–Red index in the two-season classification with the texture information. User’s accuracy was highest for the SAVI-index in the two-season image with the texture band, while it was lowest for the wet season image classification with the Green–Red index.

#### Pixel-Based

Pixel-based classification overall accuracy was highest for the Blue–Green–NIR index image for the wet season, and lowest for the Green–Red, also for the wet season. Similarly, user’s accuracy was also highest for the Blue–Green–NIR index for the wet season (0.89), and lowest (0.75) for the Green–Red index for the wet season image. Inversely, producer’s accuracy was highest for the Green–Red index for the two-season image, and lowest for the Blue–Green–NIR index for the same image.

#### Seasonal Classifications

##### Wet

Both the Green–Red and the Blue–Green–NIR indices performed comparably well for the wet season image, in terms of producer’s accuracy, while SAVI did better for the user’s accuracy, for the object-based classification. For pixel-based classification, the Green–Red index fared poorly for all three accuracy types (**Table 6**).

**Table 6:** Classification accuracies for wet season image.

	Producer’s		User’s		Overall	
	Object-based	Pixel-based	Object-based	Pixel-based	Object-based	Pixel-based
SAVI	0.494	0.494	0.886	0.813	0.609	0.574
Green–Red index	0.544	0.494	0.811	0.750	0.600	0.539
Blue–Green–NIR index	0.544	0.494	0.860	0.886	0.626	0.609

##### Two-season

Again, the Green–Red and Blue–Green–NIR indices performed well in terms of producer’s accuracy as well as overall accuracy, while the SAVI performed well in terms of user’s accuracy for the two-season image for the object-based classification (**Table 7**). For pixel-based classification results, there seems to be no clear consistent result, and the indices have performed variably for the different accuracies measured.

**Table 7:** Classification accuracies for two-season image.

	Producer’s		User’s		Overall	
	Object-based	Pixel-based	Object-based	Pixel-based	Object-based	Pixel-based
SAVI	0.481	0.506	0.905	0.870	0.609	0.609
Green–Red index	0.570	0.532	0.833	0.840	0.626	0.609
Blue–Green–NIR index	0.544	0.506	0.878	0.870	0.635	0.609

## Discussion and Conclusion

### Overall Conclusion

Object-based classification of VHR data has proved very useful in recent years for achieving specific goals in mapping landscapes. The attempt to identify *Lantana* in the understorey vegetation of tropical forests has been fairly successful in this exercise with clumps of *Lantana* being mapped out clearly, especially in the pure stands, and also in areas where they occur in the understorey of other vegetation types. Broad conclusions drawn from this study indicate that incorporating texture features and multi-season imagery definitely improves classification success. Object-based classification has proved useful in delineation of pure clumps of the invasive shrub itself, as well as in identification of classes where it occurs mixed with other species in the understorey. Multi-season VHR data can prove beneficial in marking out areas infested with invasive shrub *Lantana* when used with the appropriate techniques for mapping them in tropical forests.

### Texture

The use of the texture band in improving classification has been recorded earlier (Tsai and Chou, 2006; Xie et al., 2008), and this did indeed prove true for this exercise. The overall appearance of the classified image at the higher order classes showed more contiguous patches for the one where texture was included, as compared to the one where it was not. Accounting for spatial variation in pattern of spectral response, the texture data can add to the information provided by the spectral bands, such that target species/class may be differentiated from other surrounding classes (Kimothi and Dasari, 2010). Class accuracies in delineation of the land cover classes were not calculated, as that was not the purpose of this exercise. However, when these higher order class delineations, with texture incorporated into the input set, were further used for index-based thresholding, the accuracies for *Lantana* identification increased.

### Seasonality

Using seasonal data definitely proved beneficial in improving the class delineations for the higher order classes (**Tables 6**, **7**). Frohn et al. (2011) have also achieved minor improvements in accuracy when using multi-season images over single season images for performing segmentation of Landsat data for wetland classification. A visual inspection of both the images indicates the areas in the image that harbor vegetation types which are seasonally dry, as is also evident from the existing vegetation map that was used in the exercise. However, visually, the dry season image alone did not indicate any presence of *Lantana*, especially in the drier land cover types such as scrub and Dry Deciduous Forest. Observations in the field have shown the proliferation and abundance *of Lantana* under certain land cover types, such as Moist Deciduous Forests, while under certain other land cover types, such as Evergreen and Semi-Evergreen Forests, it is practically absent or straggling, which would be impossible to capture on RS data. The primary higher level class delineations using two season images in the object-based classification (based on the segmentation of the data), as well as the pixel-based classification were more realistic than those using only the wet season image. As a result, the last step of final delineation of *Lantana* presence areas using index thresholding proved effective with the use of two-season images, accounting for the drier areas in the second season.

### Pixel vs. Object

A major objective of this study was to compare and observe variations in the classification potential of pixel-based vs. object-based classification. Accuracy tables indicate that the object-based classification has worked better than the pixel-based, although the differences in accuracy are minimal (0–0.04). For the higher level classes, a visual inspection indicates better spatial organization of the land cover types with the object-based method. Segmentation of the two season data produced objects that were faithful to the occurrences in the field, and followed landscape variation also, such as alignment to roads or slopes, which were not so evident in the segmentation produced from the wet season image, which had many more objects identified, increasing the complexity unnecessarily. Studies in tropical areas elsewhere have also indicated the use of the object-based approach as effective, especially when different levels of objects are used together to identify the invasive species of interest (Walsh et al., 2008). Although the wet season image visually indicated presence of the shrub in certain locations, the dry season image did not indicate that from the spectral data, especially in drier forests such as those with bamboo or in the lower elevation scrub area. However, the combination of all 12 bands together helped in identifying these clusters well, especially for the pure *Lantana* class. Again, the shapes of the objects in the pure *Lantana* class obtained in the object-based classification correspond well to the shapes and sizes of the *Lantana* shrub complexes seen in the field. Pixel-based classification, however, could not identify such compact polygons conforming to the pattern on the ground, and remained somewhat scattered with other class pixels. A more efficient accuracy assessment technique for checking the accuracies of object-based classification would have been to use a pattern-based accuracy assessment technique such as that by Desclée et al. (2006).

**FIGURE 7:**
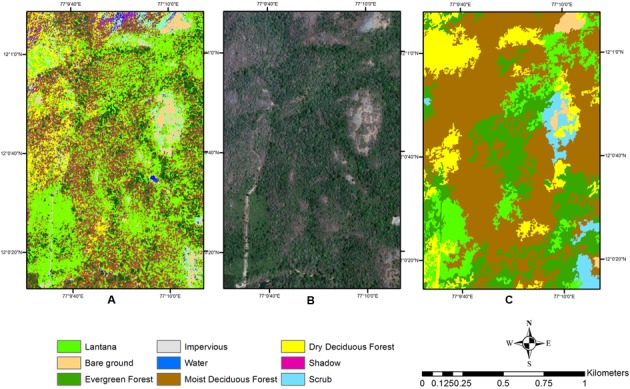
Detail of the study site comparing pixel-based and object-based classification, showing the salt-and-pepper effect in the pixel-based classification. **(A)** Pixel-based classification. **(B)** Wet season image. **(C)** Object-based classification.

**FIGURE 8:**
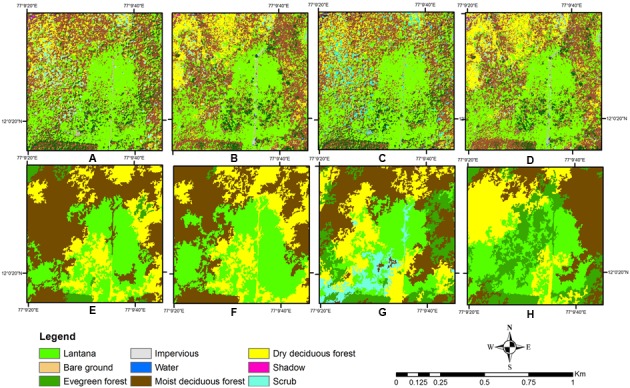
Detail of study site comparing pixel-based and object-based classification for the pure *Lantana* class. (Top row) Pixel-based classification, (Bottom row): Object-based classification. **(A,B,E,F)** Classification with texture image. **(C,D,G,H)** Classification without texture image. **(A,C,E,G)** Wet season classification. **(B,D,F,H)** Two seasons classification.

The pixel-based classification did perform similarly for the two season data, but the salt-and-pepper effect could not be eliminated even with refinement of signatures (**Figures 7**, **8**). The use of only spectral information in a parametric classifier causes every pixel to be evaluated, and hence in a VHR dataset, the spectral variability within an object may cause this effect. Other work in mixed habitat areas has yielded similar results showing reduction of salt-and-pepper effect when an object-based approach was used instead of pixel-based approach showing its strength as a contextual classifier (Yu et al., 2006).

Also, several parts of the areas near or around Evergreen forest were classified into the pure *Lantana* class, which is rarely or almost never observed in the field (Sundaram and Hiremath, 2012). Thus, this cover type was always overclassified in the pixel-based classification. The reverse was true for the Moist Deciduous forest type, which was well identified in the object-based classification, while not so clearly separated out in the pixel-based method (**Figure 7**). A comparative closer look at the two types of classification where pure *Lantana* class was identified also showed misclassifications with Scrub or Evergreen classes for certain locations (**Figure 8**). From previous reports (Sundaram, 2011; Niphadkar et al., 2016) and our field observations the Moist Deciduous forest type supports the maximum amount of *Lantana*. This was later identified clearly in the thresholding exercise.

### Indices

The indices used in this study are a subset of those tested for a good delineation of *Lantana* based on earlier studies in tropical forests. Kandwal et al. (2009) have reported the use of several indices which they tested in the Himalayan forests, and arrived at the conclusion that SAVI and its various versions proved most effective for the detection of *Lantana*. However, our classification results do not indicate a substantial improvement in detection using SAVI vs. using other indices. Spatial visualization of the maps shows that the Northwest corner of the study section was under-represented for the invasive shrub in the SAVI results, as compared to the other two indices, across all methods used. However, this area has predominantly dry forests and scrub vegetation which supports dense thickets of *Lantana* as seen in the field. The seasonality of the satellite data may have been the reason for this reduction in delineation of the shrub from this area. This drier area loses leaf for almost all the vegetation and most of the landscape supports thorny dry stems during the months of January – March. Although *Lantana* occurs in the understorey and is in leaf during early January, the leaves are not large and turgid with moisture, which may have reduced their response to the SAVI index. However, these areas were well-delineated with the other two indices, which incorporated the red band and the blue band. Combinations of blue, red, and green bands have shown to be effective for vegetation discrimination in arid landscapes (Pickup et al., 2000). Both the blue and the Red bands show higher reflectance values in drier forests as compared to moister vegetation areas, and seem to have therefore picked up the *Lantana* signal in the drier forests much better than SAVI.

### Discussion

The aim of delineating areas occupied by invasive shrub species *Lantana* was achieved in this exercise with varying precision. Other studies for *Lantana* classification using RS have reported much higher accuracies (95% Dlamini – ranches and pasture, Swaziland; 96% Kimothi et al. – Sal forest, Sivalik hills, India; 84% Kandwal et al. – Sal forest, Sivalik hills, India; 84% Taylor et al. – logged forest, Australia). However, these studies have been conducted in areas where pure stands of *Lantana* were clearly occurring alongside other vegetation types, or in logged forests where *Lantana* appeared as secondary growth in continuous stands. This study is the first of its kind where a hierarchical approach has been undertaken, to map *Lantana* at two levels – one as a pure stand, and the other as an understorey sub-class within other forest classes. Again, other studies using indices for mapping of other understorey shrubs such as *C. odorata* have acquired higher accuracies (87%), however, with a probability of occurrence approach and not a direct classification approach (Malahlela et al., 2015). Yet other studies that have managed to map understorey shrub species using an object-based, hierarchical approach on VHR aerial photo or satellite imagery data with higher accuracies have been conducted for continuous stands of the invasive species, in temperate areas with surrounding forests experiencing a distinct leaf-fall period or distinct phenological offset, which enables better separation of the invasive shrub (Mullerova et al., 2013; Shouse et al., 2013) and not in tropical areas.

Moreover, higher accuracies among studies specific to tropical understorey areas have been achieved either with small tree species and not shrubs [(Pouteau et al., 2011) – 97%; (Lu et al., 2013) – 84%; (Gavier-Pizarro et al., 2012) – 84%], or using abundance values from field data of large contiguous stands of the invasive species (Padalia et al., 2012) and not dense understorey shrubs, or with intensive field data along with multiple image datasets and active sensors such as LIDAR or RADAR [(Asner et al., 2008b) – 91%; (Ghulam et al., 2014) – 99%] which we did not have access to. Active data sensors, although very effective, are almost impossible to acquire in India due to prohibitive costs and severe security regulations on commissioning aerial data acquisition. These limitations therefore restrict the possibilities of investigating any RS studies with active data sensors in India.

This study has also opened up the possibilities of future research building on the strong capability of segmentation for object definition, by exploring various parameters for segmentation, as well as testing different segmentation algorithms. Future work can also focus on exploring vegetation indices in much further detail, as well as considering non-orthogonal indices which may prove effective in separation of shrub vs. canopy in a multi-season mode. Amongst possibilities for technique refinements, this research also suggests that given the increasing popularity and efficiency of object-based classifications, there is scope for developing some form of object-based accuracy assessments which would allow for a more faithful check on the patterns obtained from these classification procedures.

This study has helped highlight the importance of VHR data and an object-oriented approach in marking out areas infested with invasive shrub species in a tropical dry forested landscape in the Western Ghats region of southern peninsular India. The success of the object-based approach is in the fact that although the shrub is small in size, the spatial resolution of the VHR data is fine enough to characterize the shrub within several pixels, which allows for separate objects to be delineated, even with their scattered occurrence within the forest. This mapping attempt is the first of its kind for mapping the invasive shrub *Lantana* in the Western Ghats, using a hierarchical object-oriented approach with VHR data, proving the potential of this methodology in implementation among tropical tiered forests. Plant invasion is an increasing hazard in preserving natural ecosystems since many a times it may occur due to disturbances induced by humans. Management of these disturbances has to be timely, in order to prevent major spreads. This study, is therefore a step forward in developing valuable future mechanisms to monitor understorey shrubs in complex tropical forests, proving particularly effective for managing invasion at its early onset stages.

## Author Contributions

MN conceptualized part of the study, did field work, RS and statistical analysis, and wrote the manuscript. HN helped in refining conceptualization of the study, supervised the RS and field work, and co-wrote the manuscript. CT and MA helped in devising the RS methodology, and in pre-processing of the satellite data, as well as some of the mapping work. PB helped in conceptualization, facilitation and supervision of the RS work.

## Conflict of Interest Statement

The authors declare that the research was conducted in the absence of any commercial or financial relationships that could be construed as a potential conflict of interest.
