# Supplementary material for: Comparing Pixel and Object-Based Approaches to Map an Understorey Invasive Shrub in Tropical Mixed Forests
Source: Front Plant Sci. 2017 May 31;8:892. doi: 10.3389/fpls.2017.00892 (PMC5450565; doi:10.3389/fpls.2017.00892)
Supplement: Supplementary file 1 [file Table_1.DOCX]

**Supplementary Table 1. Formulae for calculating indices tested.**

| \| Index name \| Formula \| \| --- \| --- \| \| Soil Adjusted Vegetation Index \| ((NIR-RED)/(NIR+RED+L))*(1+L), where L = 1.0 \| \| Blue-Green-Near Infra Red \| (Green+((Blue/ NIR)/Green)) \| \| Green-Red \| Green-Red \| \| Normalised Difference Vegetation Index \| (NIR-Red)/NIR+Red) \| \| Transformed Vegetation Index \| SQRT (((NIR-RED)/(NIR+RED)+0.5)) \| |  |
| --- | --- | --- | --- | --- | --- | --- | --- | --- | --- | --- | --- | --- | --- |
